# Associations between serum copper, zinc, selenium level and sex hormones among 6–19 years old children and adolescents in NHANES 2013–2016

**DOI:** 10.3389/fendo.2022.924338

**Published:** 2022-09-12

**Authors:** Lishun Xiao, Chengcheng Yang, Wen Gu, Rong Liu, Ding Chen

**Affiliations:** ^1^ Department of Biostatistics, School of Public Health, Xuzhou Medical University, Xuzhou, China; ^2^ Center for Medical Statistics and Data Analysis, Xuzhou Medical University, Xuzhou, China; ^3^ Key Laboratory of Human Genetics and Environmental Medicine, Xuzhou Medical University, Xuzhou, China; ^4^ The Fifth Affiliated Hospital, Sun Yat-sen University, Zhuhai, China; ^5^ School of medical information and engineering, Xuzhou Medical University, Xuzhou, China

**Keywords:** trace elements, sex hormones, regression diagnosis, NHANES, children and adolescents

## Abstract

Copper, zinc, and selenium are essential trace elements for human and have important effects on sex hormones. There are few studies on the relationships between the three trace elements and sex hormones. Therefore, our study aimed to investigate the relationships between serum copper, zinc, selenium and testosterone, estradiol, SHBG using data from the National Health and Nutrition Examination Survey (NHANES) 2013-2016 in participants 6-19 years. 1097 participants were enrolled and stratified into male/female children and adolescents. Weighted linear regression models combined regression diagnosis were used to estimate the relationships between trace elements and sex hormones according to the different stratifications. Our results showed that copper was inversely associated with testosterone and estradiol but positively correlated with SHBG. Zinc had positive relationships with testosterone in male adolescents and female children but an inverse relationship with testosterone in female adolescents. Furthermore, a negative association was observed between zinc and SHBG. With the rise of selenium level, testosterone and estradiol were increased but SHBG was decreased. In general, this study used more standardized statistical methods to investigate the relationships between copper, zinc, selenium and testosterone, estradiol, SHBG. Further study should pay attention to some details in statistical methods.

## Introduction

Sex hormones have crucial effects on both reproductive and non-reproductive systems, and their impact on children and adolescents is complicated, related to a variety of development and puberty issues. Secondary sex traits in children and adolescents, for example, are generated by the presence of sex hormones. Furthermore, increased sex steroid release, i.e., androgen and estrogen, would trigger the pubertal growth spurt *via* both a local and a systemic action (1, 2).

Copper (Cu), zinc (Zn), and selenium (Se) are trace elements that are needed for several biological processes. Previous researches suggested that trace elements might directly affect reproductive cell function or indirectly disrupt the hypothalamic-pituitary–testicular axis, influencing the quantity of sex steroid hormones (3, 4). Because of fast development and high trace elements needs, the symptoms of trace elements deficiency are more likely to be seen in children and adolescents. Cu, Zn, and Se intake should be sufficient during childhood and adolescence to support proper growth and development (5).

There are few researches that focused on the relationships between Cu, Zn, Se and sex hormones in children and adolescents. Meanwhile, some studies did not perform complete linear regression analysis, lacking regression diagnoses. The lack of diagnosis steps may lead to non-robust results, i.e., false positive or false negative results. Therefore, we utilized a data set from the National Health and Nutrition Examination Survey (NHANES) 2013–2016 circles including the three trace elements, and serum total testosterone, estradiol and sex hormone-binding globulin (SHBG) in participants aged 6–19 years to investigate their relationships. Simultaneously we demonstrated a specific complete regression analysis combined with detailed diagnoses procedures that epidemiological or medical researchers could refer to.

## Methods

### Study design and participants

The National Health and Nutrition Examination Survey, conducted by the Centers for Disease Control and Prevention’s (CDC) National Center for Health Statistics (NCHS), is an ongoing cross-sectional survey and collects health examination data for a nationally representative sample of the residents, civilian non institutionalized U.S. population (6).

**Figure 1:**
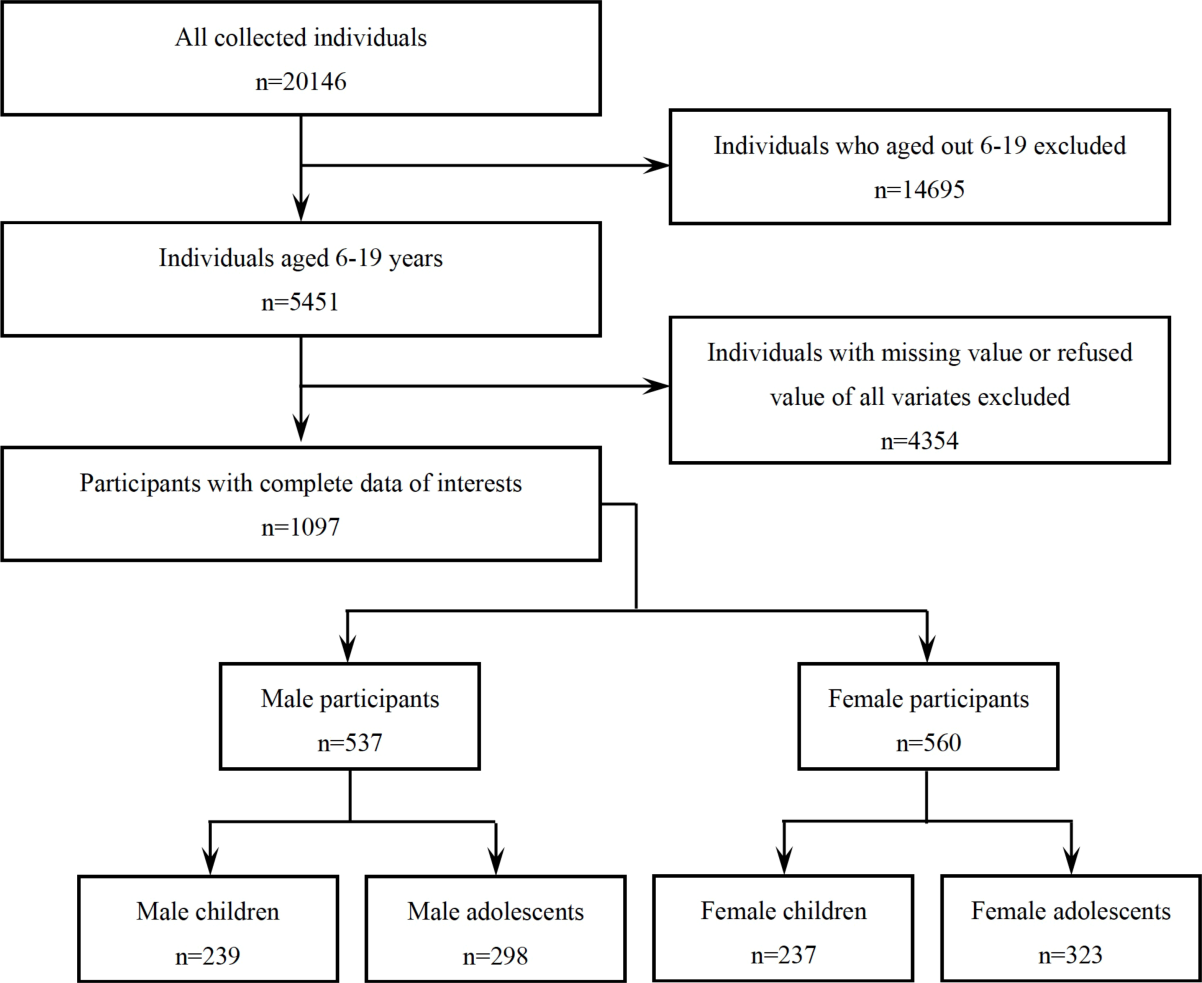
Flow chart of the participants included in this analysis (*n* = 1097), NHANES, USA, 2013–2016.

The data set of the present study were combined from two cycles of NHANES (2013–2014 and 2015–2016) with information on sex steroid hormones (serum total testosterone, estradiol and SHBG) as well as trace elements (serum Cu, Zn and Se). Our analyses were limited to 5451 participants aged 6–19 years. Of these, 4318 participants with missing or incomplete values were excluded. Finally, a total of 1097 eligible subjects with complete data of interests were included (537 males and 560 females). Children and adolescents were defined as 6-11 and 12-19 years old, respectively. The flow chart of participants was shown in **Figure 1**.

### Measurement of trace elements and sex steroid hormones

A serum multi-element inductively coupled plasma dynamic reaction cell mass spectrometry (ICP-DRC-MS) method was used to measure the entire panel of the 3 elements, Cu (μg/dL), Zn (μg/dL) and Se (μg/dL) (7). Testosterone and estradiol were measured by isotope dilution liquid chromatography tandem mass spectrometry (ID-LC-MS/MS) method. The measurement of SHBG was based on the reaction of SHBG with immuno-antibodies and chemo-luminescence measurements of the reaction products. All the detailed descriptions of the laboratory methods were referred to the Laboratory Method Files of CDC (7–9).

The lower limit of detections (LLOD) for Cu, Zn, Se, testosterone, estradiol and SHBG were 2.5, 2.9, 4.5, 0.75, 2.994 and 0.800, respectively. The proportions at or above LLOD accounted for 100%, 100%, 100%, 99.45%, 67.46% and 100%. For analytic results below LLOD, the values were substituted by $\text{LLOD}/\sqrt{2}$ .

### Covariates

Covariates were selected according to previous studies (10, 11). Some covariates were collected from demographics data of NHANES 2013–2016, including age (6–19 years), gender (male, female), race/ethnicity (Non-hispanic White, Non-hispanic black, Mexican American, other race), six-month time period when the examination was performed (November 1 through April 30, May 1 through October 31) and ratio of family income to poverty (value not greater than 1 was categorized as under poverty level, the other side was above poverty level). The BMI (kg/m^2^) levels were categorized as normal weight (less than 25), overweight (greater than or equal to 25 and less than 30) and obese (greater than or equal to 30) for subjects aged 6–19 years according to the age- and gender-specific criteria from an international study (12). Serum cotinine was included as a covariate because it reflected the status of tobacco smoke exposure. The LLOD (ng/mL) for cotinine in serum was 0.015. The values below the LLOD was placed with $\text{LLOD}/\sqrt{2}$ . Session of blood sample collection (morning, afternoon, evening) was included as a covariate because the time of venipuncture affected the fluctuations of sex steroid hormones (13).

### Statistical analysis

There existed special weights for Cu, Zn and Se because they were measured in a one-third subsample of subjects. According to the rule of “the least common denominator” in NCHS guidance, subsample weights of trace elements were adopted in our weighted analyses to treat the complex sampling design of NHANES. Since the present study combined two survey cycles (2013–2014 and 2015–2016), new special 4-year weights were determined by half of subsample weights following the NHANES tutorials (14). Considering some potential effects of age and gender, weighted analyses were separately conducted for all subjects and four subgroups, i.e., male children (6–11 years), male adolescents (12–19 years), female children (6–11 years) and female adolescents(12–19 years), respectively. Data analyses were conducted with R version 4.1.0. Some implementations were publicly available on The Comprehensive R Archive Network as an R package titled “car”. The two-tailed significance level 0.05 was considered in all analyses.

Descriptive statistics of continuous variables and proportions of categorical variables were calculated by weighted case. All weighted continuous variables of the present study did not follow normal distribution *via* Kolmogorov-Smirnov’s test. Mean and standard deviation were used to describe characteristics of continuous variables if they were approximate symmetric, otherwise median and interquartile range (IQR) were used. Data of continuous variables could be seen as approximate symmetry if its absolute value of weighted skewness (see supplementary) was not greater than 1, otherwise distributed with a skew. Demographic characteristics were compared across the four subgroups by weighted analysis of variance (ANOVA) for continuous variables and weighted Chi-square test for categorical variables. Levene’s test was used to test the homoscedasticity across the four subgroups. Welch’s ANOVA was applied when the approximate symmetry and heteroscedasticity existed among the four subgroups. A Student-Newman-Keuls (SNK) *post hoc* test was utilized to determine the differences between each two groups.

The sample weight in NHANES can be interpreted as the number or frequency of persons in the population represented by the individual (15). Solving a linear regression model constructed by data with frequency *via* ordinary least square (OLS) method was formally equivalent to a weighted linear regression *via* weighted least square method, where the weight was the frequency, ignoring the probability sense of weights. Therefore, the associations of trace elements (Cu, Zn and Se) with sex steroid hormones (testosterone, estradiol and SHBG) were estimated by weighted linear regression models. Ratio of family income to poverty, BMI and serum cotinine were handled as continuous variables in regressions. All the regression diagnoses were based on the weighted linear regressions and the detailed procedures were as follows. The three sex steroid hormones, respectively, were modeled as a function of the three trace elements adjusting for covariates across all subjects and the four subgroups. Then three kinds of extreme values, namely outliers, high leverage points and influential points, were detected after regressions (16). Outliers were points that had unusual residuals. Outliers were identified if the absolute studentized residual was significant *via* Bonferroni adjusted *P* value. Points that had high leverage meant that they had an unusual combination of independent variable values. High leverage points were identified if the hat statistic was greater than 3*p*/*n*, where *p* was the number of parameters estimated in the regression model including the intercept and *n* was the sample size. Influential points had a disproportionate impact on the values of the model parameters. They were determined when Cook’s distance was greater than 4/(*n* – *k* – 1), where *n* was the sample size and *k* was the number of independent variables. After the extreme values were removed the normality, homoscedasticity and independence of residuals were detected for weighted linear regressions. Durbin-Watson’s test was adopted to check the independence and significant results of dependence were not found. When models did not meet the normality or homoscedasticity assumptions, the Box-Cox’s transformation was applied to improve or correct the situation. The transformations of square root and natural logarithm type were employed in our models. After that, the *F*-statistics in regressions were adopted to determine whether the linearity between the dependent variable and independent variables existed and all *P* values were significant, which meant that the linearity assumptions were fulfilled. Finally, multicollinearity was detected using the statistic variance inflation factor (VIF). All VIF values of variables in all models were not greater than 4, which indicated that there existed no multicollinearity problems. Furthermore, the events per variable (EPV) principle was also fulfilled because 13 independent variables, including dummy variables, were incorporated into our models and the sample size was greater than 130 in each model (17).

## Results

### Characteristics of subjects

Descriptive statistics for demographic characteristics, trace elements and sex steroid hormones were presented in **Tables 1** and **2**. The averaged age of participants was nearly 13 years. Serum Cu levels were higher in male children (123.50 μg/dL) than in adolescents (98.53 μg/dL), but the levels in female children (118.12 μg/dL) and adolescents (116.42 μg/dL) were similar. The averaged levels of Zn and Se were 81.87 μg/dL and 122.22 μg/dL among participants. In addition, the levels of testosterone and estradiol were higher in adolescents while SHBG was just the opposite.

**Table 1:** Demographic characteristics in different subgroups.

	Total	Male		Female		P values
		Children	Adolescents	Children	Adolescents	
*n* (%)	1097 (100)	239 (21.79)	298 (27.16)	237 (21.60)	323 (29.44)	
*N* (%)	38600094 (100)	7468190 (19.35)	11926998 (30.90)	6947749 (18.00)	12257157 (31.75)	
Age (years), mean (SD)	12.79 (3.79)	8.54 (1.64)	15.15 (2.23)c***	8.70 (1.75)d***	15.39 (2.10)c***,e***	<0.001^a^
Race/ethnicity, *N* (%)						
Non-Hispanic White	20735553 (53.72)	4098602.3 (54.88)	6695143.3 (56.13)	3409718.6 (49.08)	6532088.6 (53.29)	<0.001
Non-Hispanic Black	4470180 (11.58)	867816.9 (11.62)	1460253.9 (12.24)	766690.6 (11.04)	1375419.0 (11.22)	
Mexican American	6126108 (15.87)	1141425.9 (15.28)	1776932.6 (14.90)	1423325.8 (20.49)	1784424.1 (14.56)	
Other Race	7268253 (18.83)	1360345.2 (18.22)	1994668.1 (16.72)	1348013.8 (19.40)	2565225.6 (20.93)	
Family PIR, *N* (%)						
PIR<=1	9112689 (23.61)	1770793 (23.71)	2500823 (20.97)	1959315 (28.20)	2881758 (23.51)	<0.001
PIR >1	29487405 (76.39)	5697398 (76.29)	9426174 (79.03)	4988434 (71.80)	9375399 (76.49)	
Serum cotinine, *N*(%)^b^						
<LLOD	14073182 (36.46)	2612187 (34.98)	3416099 (28.64)	3034115 (43.67)	5010780 (40.88)	<0.001
>=LLOD	24526912 (63.54)	4856003 (65.02)	8510899 (71.36)	3913633 (56.33)	7246377 (59.12)	
BMI category, *N* (%)						
Normal weight	29149987 (75.52)	6856528.4 (91.81)	8807098.2 (73.84)	6262098.2 (90.13)	7224262.1 (58.94)	<0.001
Overweight	5213428 (13.51)	381018.3 (5.10)	1706794.2 (14.31)	467642.4 (6.73)	2657973.5 (21.69)	
Obese	4236679 (10.98)	230643.6 (3.09)	1413105.5 (11.85)	218008.2 (3.14)	2374921.8 (19.38)	
Survey time period						
Nov. 1 to Apr. 30	17545321 (45.45)	3559588 (47.66)	5941689 (49.82)	2951352 (42.48)	5092692 (41.55)	<0.001
May 1 to Oct. 31	21054773 (54.55)	3908602 (52.34)	5985309 (50.18)	3996397 (57.52)	7164465 (58.45)	
Blood collection, *N* (%)						
Morning	16730845 (43.34)	2458553 (32.92)	5235360 (43.90)	3033536 (43.66)	6003396 (48.98)	<0.001
Afternoon	14155481 (36.67)	3306334 (44.27)	4585101 (38.44)	2304349 (33.17)	3959697 (32.31)	
Evening	7713768 (19.98)	1703303 (22.81)	2106537 (17.66)	1609864 (23.17)	2294064 (18.72)	

Sampling weights were applied for calculation of demographic descriptive statistics and N represents the weighted sample size.

a: Age differences of four subgroups (male children, male adolescents, female children and female adolescents) were tested by Welch’s ANOVA because the four subgroups satisfy approximate symmetry and heteroscedasticity; other differences across four subgroups were tested by Chi-square test.

b: The LLOD of serum cotinine was set at 0.015 ng/mL.

c, d, and e represent the post hoc result of SNK method with the group of Male Children, Male Adolescents, and Female Children, respectively, with ***P<0.001.

**Table 2:** Descriptive statistics for trace elements and sex steroid hormones in different subgroups.

	Total	Males		Females		*P* values
		Children	Adolescents	Children	Adolescents	
*n* (%)	1097 (100)	239 (21.79)	298 (27.16)	237 (21.60)	323 (29.44)	
*N* (%)	38600094 (100)	7468190 (19.35)	11926998 (30.90)	6947749 (18.00)	12257157 (31.75)	
Cu (μg/dL); mean (SD)	112.57 (26.13)	123.50 (22.09)	98.53 (20.22)^a***^	118.12 (21.70)^a**,b***^	116.42 (29.86)^a***,b***^
Se (μg/L); mean (SD)	122.22 (14.10)	119.48 (11.90)	124.80 (15.09)^a***^	117.07 (13.10)^b***^	124.31 (13.81)^a***,c***^	<0.001
Zn (μg/dL); mean (SD)	81.87 (14.75)	79.98 (13.38)	84.38 (16.54)^a**^	81.38 (14.56)^b**^	80.87 (13.44)^b**^	<0.001
Serum sex hormones						
TT (ng/dL); median (IQR)	23.20 (198.79)	3.61 (3.54)	376 (304.00)^a***^	5.67 (7.86)^b***^	24.00 (14.50)^b***^	<0.001
E2 (pg/mL); median (IQR)	15.90 (32.48)	2.114 (0.003)	18.40 (15.50)^a***^	2.117 (10.88)^a**^	50.80 (69.70)^a***,b***,c***^	<0.001
SHBG (nmol/L); median (IQR)	55.53 (59.73)	101.8 (68.43)	36.91 (25.83)^a***^	80.56 (61.6)^a***,b***^	51.81 (47.26)^a***,b***,c***^	<0.001

Sampling weights were applied for calculation of demographic descriptive statistics and N represents the weighted sample size. TT and E2 represent testosterone and estradiol, respectively.

Differences of four subgroups were tested by Welch’s ANOVA because the four subgroups satisfy approximate symmetry and heteroscedasticity.

a, b, and c represent the post hoc result of SNK method with the group of Male Children, Male Adolescents, and Female Children, respectively, with **P< 0.01 and ***P<0.001.

### Regression results

Regression results for sex steroid hormones (testosterone, estradiol and SHBG) and trace elements (Cu, Zn and Se) were listed in **Table 3**. For readers’ convenience, regression coefficients were denoted by $b_{j}^{i}$ , where *i* = *Cu*, *Zn*, *Se* represented Cu, Zn and Se, *j* = 0, 1, 2, 3, 4 represented the all subjects, male children, adolescents, female children, and female adolescents, *b* = *T*, *E*, *S* represented testosterone, estradiol and SHBG, respectively. And we use $\sqrt{b_{j}^{i}}$ and *ln* $b_{j}^{i}$ to represent the square root and natural logarithm transformations for the dependent variable, respectively. All participants, male adolescents, female children, and female adolescents showed a inverse association between serum Cu levels and testosterone ( $\sqrt{T_{0}^{Cu}}=-0.021$ , $T_{2}^{Cu}=-5.169$ , $\sqrt{T_{3}^{Cu}}=-0.007$ , *ln* $T_{4}^{Cu}=-0.006$ ; 95% CIs, -0.024 to -0.017, -6.092 to -4.247, -0.012 to 0.005, and -0.008 to -0.004; *P*< 0.001). Across gender and age groups, serum Cu levels were adversely linked with estradiol ( $E_{1}^{Cu}=-0.019,\;E_{2}^{Cu}=-0.207$ , *ln* $E_{3}^{Cu}=-0.016$ , $\sqrt{E_{4}^{Cu}}=-0.021$ ; 95% CIs, -0.027 to -0.010, -0.249 to -0.165, -0.023 to -0.010, and -0.031 to -0.010; *P<* 0.001).* A* positive correlation was found between serum Cu level and SHBG ( $\sqrt{S_{0}^{Cu}}=0.023$ ; 95% CI, 0.019 to 0.027; *P*< 0.001), among all subjects. However, no correlation was found between serum Cu levels and SHBG in gender or age stratification groups.

Table 3Associations between sex hormones and trace elements.

**Table:** 

		Total^a^ (*n*=519, *N*=13312147)		MC (*n*=143, *N*=3708963)		MA (*n*=222, *N*=7110701)		FC^a^ (*n*=151, *N*=3154763)		FA^b^ (*n*=192, *N*=5377366)	
		Estimate (95%)	*P* values	Estimate (95%)	*P* values	Estimate (95%)	*P* values	Estimate (95%)	*P* values	Estimate (95%)	*P* values
TT	Cu	-0.021 (-0.024, -0.017)	<0.001	-0.005 (-0.015, 0.005)	0.313	-5.169 (-6.092,-4.247)	<0.001	-0.007 (-0.012,-0.005)	<0.001	-0.006 (-0.008, -0.004)	<0.001
	Zn	-0.003 (-0.008, 0.003)	0.390	0.014 (-0.002, 0.029)	0.081	1.544 (0.269, 2.818)	<0.05	0.009 (0.002,0.017)	<0.05	-0.005 (-0.008, -0.002)	<0.05
	Se	0.018 (0.012, 0.023)	<0.001	0.021 (0.005, 0.037)	<0.05	2.984 (1.702, 4.266)	<0.001	0.002 (-0.005, 0.010)	0.516	-0.001 (-0.003, 0.002)	0.559

**Table:** 

		Total^b^ (*n* = 1097, *N*=38600094)		MC (*n* =239, *N* =7468190)		MA (*n* =229, *N* =7590013)		FC^b^ (*n*=237, *N*=6947749)		FA^a^ (*n*=185, *N*=5106113)	
		Estimate (95%)	*P* values	Estimate (95%)	*P* values	Estimate (95%)	*P* values	Estimate (95%)	*P* values	Estimate (95%)	*P* values
E2^c^	Cu	-0.019 (-0.022, -0.017)	<0.001	-0.019 (-0.027, -0.010)	<0.001	-0.207 (-0.249, -0.165)	<0.001	-0.016 (-0.023, -0.010)	<0.001	-0.021 (-0.031, -0.010)	<0.001
	Se	-0.002 (-0.007, 0.004)	0.562	0.012 (-0.004, 0.028)	0.1328	0.033 (-0.030, 0.095)	0.307	-0.001 (-0.012, 0.010)	0.8183	-0.012 (-0.035, 0.011)	0.315
	Zn	0.0128 (0.008, 0.018)	<0.001	0.006 (-0.001, 0.001)	0.479	0.150 (0.095, 0.206)	<0.001	0.009 (-0.002, 0.020)	0.108	-0.028 (-0.048, -0.005)	<0.05

**Table:** 

		Total^a^ (*n*=670, *N*=16712730)		MC^a^ (*n*=180, *N*=4591930)		MA^a^ (*n*=216, *N*=7060841)		FC (*n*=178, *N*=3952432)		FA (*n*=153, *N*=3593836)	
		Estimate (95%)	*P* values	Estimate (95%)	*P* values	Estimate (95%)	*P* values	Estimate (95%)	*P* values	Estimate (95%)	*P* values
SHBG	Cu	0.023 (0.019, 0.027)	<0.001	0.008 (-0.001, 0.016)	0.0759	0.003 (-0.004, 0.010)	0.435	0.111 (-0.032, 0.253)	0.127	-0.053 (-0.150, 0.045)	0.287
	Se	-0.002 (-0.010, 0.005)	0.557	-0.019 (-0.034, -0.003)	<0.05	0.009 (-0.001, 0.017)	0.061	0.260 (0.004, 0.516)	<0.05	-0.084 (-0.225, 0.058)	0.245
	Zn	-0.020 (-0.026, -0.012)	<0.001	0.004 (-0.010, 0.019)	0.558	-0.014 (-0.022, -0.006)	<0.05	-0.026 (-0.293, 0.240)	0.846	-0.229 (-0.355, -0.104)	<0.001

TT and E2 represent testosterone and estradiol, respectively. n and N represent unweighted and weighted sample size included in models, respectively. MC, MA, FC and FA represent male children, male adolescents, female children and female adolescents, respectively.

All models were adjusted for: age, race/ethnicity (non-hispanic white, non-hispanic black, mexican american, other race), ratio of family income to poverty, serum cotinine, BMI, six month time period when surveyed (November 1 through April 30, May 1 through October 31), session of blood sample collection (morning, afternoon, evening).

a: Square root transformation was adopted.

b: Natural logarithm transformation was adopted.

c: The all subjects, male children, female children groups did not remove extreme values because only two values (2.114, 2.117) of estradiol were retained after removing extreme values. These two values were obtained by $\text{LLOD}/\sqrt{2}$ .

Male adolescents and female children had positive associations between serum Zn levels and testosterone ( $T_{2}^{Zn}=1.544,\;\sqrt{T_{3}^{Zn}}=0.009$ ; 95% CIs, 0.269 to 2.818 and 0.002 to 0.017; *P <*0.05 and <0.05; respectively). Regardless of gender or age, changes in estradiol related to serum Zn levels are not statistically significant. In the children stratification group, there existed associations between serum Zn levels and SHBG, but the association was negative for male and positive for female ( $\sqrt{S_{1}^{Zn}}=-0.019,\;S_{3}^{Zn}=0.260$ , 95% CIs, -0.034 to -0.003, 0.004 to 0.516; *P*< 0.05). The relationship between SHBG and Zn was not statistically significant across adolescents.

Serum Se levels were positively correlated with testosterone and estradiol except for female adolescents. Male participants had statistically significant associations between serum Se levels and testosterone ( $T_{1}^{Se}=0.021\text{ and }T_{2}^{Se}=2.984$ ; 95% CIs, 0.005 to 0.037 and 1.702 to 4.266; *P* < 0.05 and <0.001; respectively). Furthermore, there existed statistically significant positive associations between serum Se levels and estradiol in male adolescent group and the negative association in female adolescent group ( $E_{2}^{Se}=0.15,\;\sqrt{E_{4}^{Se}}=-0.028$ ; 95% CIs, 0.095 to 0.206 and -0.048, -0.005; *P*< 0.001 and <0.05; respectively). Male and female adolescents’ SHBG levels had a negative connection with serum Se levels $(\sqrt{S_{2}^{Se}}=-0.014,\;\sqrt{S_{4}^{Se}}=-0.229$ ; 95% CIs, -0.022 to -0.006, -0.355, -0.104; *P*< 0.05, *P*< 0.001; respectively). Male and female children had no statistically significant associations between serum Se and SHBG.

## Discussion

The current cross-sectional study was based on the data of NHANES 2013–2016 with the purpose of investigating the relationships between trace elements (Cu, Zn, Se) and sex steroid hormones (testosterone, estradiol, and SHBG) among male and female children and adolescents. Our results showed that serum Cu levels was inversely associated with testosterone and estradiol, but positively correlated with SHBG. Serum Zn levels had a positive relationship with testosterone in male adolescents and female children but an inverse relationship with testosterone in female adolescents. Furthermore, relationships between serum Zn levels and SHBG in male children and femle children groups were opposite. Finally, testosterone and estradiol elevated with the increase of serum Se levels, but SHBG was the opposite.

Cu is a necessary element and harmful in high doses (18). Cu could stimulate ROS creation and bind directly to the thiol groups of cysteine, causing oxidation to reduce the production of sex hormones (19). Additionally, Cu has an xenoestrogenic effect on the oestradiol feedback pathway, which is assumed to create a negative feedback loop in the hypothalamus-pituitary-gonadal axis, lowering testosterone and estradiol production (20). Our results showed that serum Cu levels was inversely associated with testosterone and estradiol, but positively correlated with SHBG. These results are consistent with previous studies. One study based on the Flemish Environment and Health study (21) showed that Cu was negatively associated with testosterone and estradiol with significance in male adolescents. Another longitudinal cohort study of Mexico proved that Cu was inversely related to testosterone among female children (22). Our results of SHBG were shown to be positively associated with serum Cu levels overall while this association was found only in male adolescents in the Flemish study (23).

Zn involved in sex hormone synthesis, sexual maturity, and androgen metabolism. All of those linked to the development of the main and secondary sex organs in the male and all phases of the reproductive process in the female. Zinc deficiency impairs angiotensin converting enzyme (ACE) function, resulting in low testosterone levels, poor sperm quality, and a greater rate of male infertility (24). In the current study, serum Zn concentrations were shown to be positively connected to testosterone production in male adolescents and female children, but adversely related to testosterone levels in female adolescents. One study among puberty gynecomastia males had also reported the similar results (25). Another study suggested medicinal doses supplementation of Zn might increase testosterone level (26, 27). In addition, our results showed that serum SHBG level was inversely related to Zn level overall. However (28), discovered the negative correlation only in males.

Se functions as an antioxidant as well as a peroxynitrite scavenger. It is the primary component of glutathione peroxidase, which reduces free radical production and lipoprotein peroxidation (29). Many biochemical studies have provided the strong rationales for the concept that selenium status also interacts with sex hormones secretion (30). Yun Liu, et al. (31) discovered that insufficient Se consumption might be related to delayed pubertal sexual development in 10–18 years old males but not in females, implying a sex-specific trend. In our study, Se was shown to be positively associated with testosterone in children and adolescents. However, the positive association was shown only in male adolescents in the study using NHANES data of 2011–2012 (32). Additionally, adult research showed that Se supplementation could increase testosterone levels in infertile men (33), and estradiol level of females who lived in high Se area was higher than that in areas with normal Se (34).

A significant strength of this study is that we manipulated the diagnosis procedure of regression models to ascertain whether the models satisfied the following assumptions: (1) normality of residuals; (2) linearity between response and independent variables; (3) homoscedasticity of different groups of residuals; (4) independence of residuals; (5) no collinearity between independent variables. Under the last four assumptions, Gauss-Markov's theorem (35) ensures that the resulting estimators of regression coefficients are uniformly minimum-variance unbiased estimators. The normality of residuals insured the accuracy of hypothesis test of estimators, especially under the situation of a small sample size. Therefore, the estimators and the corresponding *P* values were more accurate, thereby the association results were more accurate. The normality assumption can be neglected in the case of large samples because of the central limit theorem. Without regression diagnosis, we cannot judge whether the resulting estimators is good or bad. To the best of our knowledge, there were considerable numbers of studies ignore the importance of regression diagnosis steps, which might induce inaccurate *P* values even false positive or false negative results. The sign and *P* values of estimators are crucial criteria to judge whether the associations make sense. Therefore, the accuracy of estimators and *P* values must be guaranteed and the diagnosis procedures must be implemented after regressions.

The present study also has some limitations. Firstly, this cross-sectional research limited the inferences based on the present results. Moreover, because only two values of estradiol (2.114, 2.117) remained after removing extreme values during the diagnosis of regression models for overall, male children and female children groups, raw data of them were used for regression analysis, which might have an impact on the results. Lastly, after Box-Cox’s transformation the estimators of regression coefficients were slightly complex, especially the square root transformation. For the natural logarithm transformation, the averaged value of the dependent variable *y* will add $\left(e^{\hat{\beta }}-1\right)\times 100\%$ when the independent variable *x* adds one unit, where $\hat{\beta }$ is the estimator of the coefficient of *x*. Whereas, the square root transformation cannot obtain a similar specific relationship.

In conclusion, the present study suggested the relationships between trace elements (Cu, Zn and Se) and three sex hormones (testosterone, estradiol and SHBG) in NHANES 2013–2016. Compared with other similar studies, we used more standardized statistical methods. Many epidemiological studies tend to focus on the relationships between observation variables, while neglecting some details in statistical methods, which should be paid attention to in future research.

## Data availability statement

The data set presented in this study can be found in online repositories of NHANES. The data file names can be found at:DEMO_H (https://wwwn.cdc.gov/nchs/nhanes/search/datapage.aspx?Component=Demographics&Cycle=2013-2014);BMX_H (https://wwwn.cdc.gov/nchs/nhanes/search/datapage.aspx?Component=Examination&Cycle=2013-2014); COT_H, CUSEZN_H, FASTQX_H and TST_H (https://wwwn.cdc.gov/nchs/nhanes/search/datapage.aspx?Component=Laboratory&Cycle=2013-2014); DEMO_I (https://wwwn.cdc.gov/nchs/nhanes/search/datapage.aspx?Component=Demographics&Cycle=2015-2016); BMX_I (https://wwwn.cdc.gov/nchs/nhanes/search/datapage.aspx?Component=Examination&Cycle=2015-2016); COT_I, CUSEZN_I, FASTQX_I and TST_I (https://wwwn.cdc.gov/nchs/nhanes/search/datapage.aspx?Component=Laboratory&Cycle=2015-2016).

## Ethics statement

This study was reviewed and approved by We used deidentified publicly accessible datasets, and this study was considered exempt from the Institutional Review Board. Written informed consent to participate in this study was provided by the participants’ legal guardian/next of kin.

## Author contributions

Conceptualization, RL, DC and CY. Methodology, LX. Software, LX. Validation, CY, RL and WG. Formal analysis, LX. Data curation, LX and DC. Writing—original draft preparation, CY. Writing—review and editing, RL. Visualization, LX. Supervision, RL and DC. Project administration, RL and DC. All authors contributed to the article and approved the submitted version.

## Funding

LX is funded by the National Natural Science Foundation of China (No. 12001470) and the China Postdoctoral Science Foundation (No. 2020M671607). CY is funded by the Zhuhai Science and Technology Program (No. ZH22036201210134PWC). RL is funded by 2021 High-Level Innovative and Entrepreneurship Talent Program, Jiangsu Province (No. JSSCBS20211260). DC is funded by the Research Initiation Foundation of Xuzhou Medical University (No. D2017018).

## Acknowledgments

We greatly appreciate the reviewers whose comments and suggestions helped improve this article.

## Conflict of interest

The authors declare that the research was conducted in the absence of any commercial or financial relationships that could be construed as a potential conflict of interest.

## Publisher’s note

All claims expressed in this article are solely those of the authors and do not necessarily represent those of their affiliated organizations, or those of the publisher, the editors and the reviewers. Any product that may be evaluated in this article, or claim that may be made by its manufacturer, is not guaranteed or endorsed by the publisher.
