# Supplementary material for: Associations between serum copper, zinc, selenium level and sex hormones among 6–19 years old children and adolescents in NHANES 2013–2016
Source: Front Endocrinol (Lausanne). 2022 Sep 12;13:924338. doi: 10.3389/fendo.2022.924338 (PMC9511025; doi:10.3389/fendo.2022.924338)
Supplement: Supplementary file 1 [file Table_1.docx]

Supplementary Material

# Supplementary Table

Table S1. Weighted skewness of continuous variables

|  | Total | Males | | Females | |  |
| --- | --- | --- | --- | --- | --- | --- |
|  |  | Children | Adolescents | Children | Adolescents |  |
| Age (years) | 0.01 | 0.02 | 0.12 | -0.11 | 0.08 | |
|  | -0.15 | 0.07 | 0.25 | -0.21 | -0.01 | |
| Selenium (ug/L) | 0.42 | 0.63 | 0.26 | 0.25 | 0.45 | |
|  | 0.42 | 0.55 | 0.32 | 0.11 | 0.51 | |
| Copper (ug/dL) | 1.09 | 0.31 | 0.67 | 0.80 | 1.88 | |
|  | 1.17 | 0.51 | 0.58 | 0.63 | 1.80 | |
| Zinc (ug/dL) | 0.41 | 0.30 | 0.51 | 0.35 | 0.26 | |
|  | 0.54 | 0.26 | 0.75 | 0.48 | 0.09 | |
| Testosterone (ng/dL) | 1.76 | 5.14 | 0.06 | 1.96 | 3.78 | |
|  | 1.66 | 4.47 | 0.16 | 1.97 | 3.28 | |
| Estradiol (pg/mL) | 3.37 | 4.77 | 0.54 | 5.39 | 1.81 | |
|  | 3.51 | 4.81 | 0.59 | 5.69 | 1.96 | |
| SHBG (nmol/L) | 1.71 | 0.67 | 1.94 | 0.99 | 3.28 | |
|  | 2.02 | 0.79 | 1.83 | 0.80 | 2.97 | |
